# Barriers Affecting Diabetic Foot Care in Primary Healthcare Settings in Al-Ahsa, Saudi Arabia

**DOI:** 10.7759/cureus.90717

**Published:** 2025-08-22

**Authors:** Hanan S Alabdulmuhsin, Huda M Alethan, Fatimah J Al-Shehab

**Affiliations:** 1 Family Medicine, Ministry of Health, Al-Ahsa Health Cluster, Al-Ahsa, SAU

**Keywords:** barriers, diabetic foot, knowledge, practice, saudi arabia

## Abstract

Introduction and aim: Diabetes mellitus is a metabolic disease characterized by hyperglycemia, leading to long-term organ damage and complications like foot ulcers. Aggressive management of diabetic foot ulcers can prevent complications and lower extremity amputations. This study aimed to assess the barriers faced by physicians in managing diabetic feet in primary care settings.

Methods: This is a descriptive cross-sectional study conducted in primary healthcare settings in Al-Ahsa, Saudi Arabia, between 2024 and 2025, involving 220 participants through simple random sampling. The study used a structured, valid, pretested, and self-administered questionnaire to collect data. The Statistical Package for the Social Sciences (SPSS) version 27 (Armonk, NY: IBM Corp.) was used to analyze the data. Both descriptive and inferential statistics were used, with a p≤0.05 considered statistically significant.

Results: This study included 220 physicians (mean age: 32.14±6.05 years, 53.6% male), 130 (59.0%) had practiced for less than five years, 97 (44.1%) reported less than five continuing medical education (CME) hours related to diabetes, and 132 (60.0%) reported no special training in diabetes mellitus (DM). A majority of them, 160 (72.7%), rated their knowledge as average; 197 (89.5%) reported educating patients on preventive foot care, and 189 (85.9%) reported recommending therapeutic footwear. Only 102 (46.4%) probe for bone exposure in open diabetic foot (DF) infections, and 93 (42.3%) request serial radiographs. More than half identified a lack of continuing education and knowledge as the main barriers to the effective diagnosis and management of diabetic foot infections. Further barriers reported by participants were the absence of a vascular medicine specialty and a lack of diabetic foot management guidelines. Furthermore, 140 (63.6%) reported a lack of access to foot examination tools, and 152 (69.1%) reported insufficient time for comprehensive foot examinations.

Conclusion: This study highlights significant gaps in knowledge, practice, and resources among healthcare professionals involved in diabetic foot management. The findings demonstrate the need for targeted interventions in Al-Ahsa to improve clinician competency, enhance resource availability, and address systemic challenges.

## Introduction

Diabetes mellitus is defined by the American Diabetes Association (ADA) as a group of metabolic diseases characterized by hyperglycemia, this elevation of blood glucose level is caused by defects in insulin secretion, insulin action, or both [1]. It is considered one of the most common diagnoses made by family physicians [2]. The 10th edition of the International Diabetes Federation (IDF) Diabetes Atlas reported a continued global increase in diabetes prevalence. There are 537 million adults worldwide living with diabetes. This number is predicted to rise to 643 million by the year 2030. In the Middle East and North Africa, it has been reported that one in six adults is living with diabetes, with the number of people having diabetes being 73 million. Saudi Arabia is considered one of the top five countries in the Middle East and North Africa with the highest number of people with diabetes. In 2011, the number was 2.8 million diagnosed cases of diabetes in Saudi Arabia; this number increased to 4.3 million in 2021 [3].

The chronic elevation of blood glucose levels in diabetes can lead to long-term damage, dysfunction, and failure of multiple body organs [1]. One of these complications is a foot ulcer, which is considered a significant complication of diabetes that precedes lower extremity amputation [4]. Diabetic foot ulcer is a major source of preventable morbidities in diabetic patients. The morbidity following ulceration is high, with lifetime lower extremity amputation of 20% and 50-70% five-year mortality [5].

The burden of diabetic foot complications is restricted not only to the patient's quality of life but also to the healthcare facilities, which incur high costs during admission time, investigations, procedures, medications, and dressings [6]. Thus, aggressive management of diabetic foot ulcers often prevents such complications and reduces the incidence of lower extremity amputations. This can be achieved by multidisciplinary management programs that aim to prevent, educate, and regularly examine the feet of diabetic patients [4].

There are several barriers to a proper foot care service. Some of these barriers arise from the perspectives of practitioners [7]. One study conducted at a conference in the United Kingdom demonstrated that the crucial barrier to diabetic foot care was the delay in accessing specialist care. This issue must be addressed to achieve optimum patient care [8]. Furthermore, early referral to podiatry clinics is recommended [9].

Diabetic foot is an important complication of diabetes that can be prevented with early recognition. In primary healthcare in Al-Ahsa, Saudi Arabia, the absence of a podiatry clinic in the primary healthcare center (PHC) setting poses a significant barrier to the early recognition of diabetic foot. Furthermore, the lack of specialties to refer to is thought to be a barrier to diabetic foot care [10].

The scarcity of vascular surgeons in Al-Ahsa presents a considerable barrier to effective diabetic foot management. Furthermore, a lack of readily available guidelines concerning the resources required for diabetic foot screening and management exacerbates this challenge. Compounding these issues, inadequately trained staff can significantly impede diabetic foot screening initiatives. Addressing these barriers is crucial for improving screening rates and preventing diabetic foot complications. This study aimed to assess the barriers encountered by physicians in managing diabetic foot within primary healthcare center (PHC) settings in Al-Ahsa.

This study sought to answer the following research question: What are the barriers encountered in providing effective diabetic foot care among primary healthcare professionals in Al-Ahsa, Saudi Arabia? The hypothesis proposed that the primary healthcare professionals in Al-Ahsa face significant barriers, which negatively impact the quality of diabetic foot care they provide.

## Materials and methods

Study design and settings

This descriptive cross-sectional study was conducted in primary healthcare settings in Al-Ahsa, Saudi Arabia, from September 5, 2024, to May 15, 2025. The study included all physicians working in primary healthcare in Al-Ahsa, specifically general practitioners, family medicine specialists, family medicine consultants, and family medicine residents. The inclusion criteria consisted of all physicians working in primary healthcare in Al-Ahsa, which included general practitioners, family medicine specialists, family medicine consultants, and family medicine residents. We excluded doctors who declined to participate in this study.

According to the Al-Ahsa health cluster, the total number of physicians working in PHC settings in Al-Ahsa is 500, including 91 family medicine residents. To calculate the sample size for our study, we used the Steven Thompson formula [11].

$n = \frac{N \times p(1 - p)}{\left[\frac{(N - 1) \times d^2}{z^2} \right] + p(1 - p)}$

Where n is the sample size, N is the population size (500), Z is the Z-value corresponding to the desired confidence level (1.96 for 95% confidence), p is the estimated proportion of the population with the attribute of interest (often assumed to be 0.5 for maximum sample size), and e is the margin of error (0.05 for 5%). Using the Steven Thompson formula, the sample size was calculated as follows.

$n = \frac{500 \times (1.96)^2 \times 0.5(1 - 0.5)}{(0.05)^2 \times (500 - 1) + (1.96)^2 \times 0.5(1 - 0.5)} = 218$

Thus, the study included 220 participants, selected through simple random sampling using an Excel sheet generated from the sampling frame.

Study instrument

Data were collected using a structured, valid, pretested, and self-administered modified questionnaire from a previous similar study, with permission from the author to use the questionnaire [10]. The questionnaire included three sections. The first section was about demographic data. The second section evaluated the knowledge, attitude, and practice of physicians. The last part assessed the barriers and challenges (tables in appendix). To ensure the reliability of the research instrument used in this study, Cronbach's alpha was calculated, and the questionnaire was revised by three experts in family medicine and epidemiology. In this study, Cronbach's alpha was found to be 0.90, which signifies a high level of reliability.

Data analysis

Data were analyzed using the Statistical Package for the Social Sciences (SPSS) version 27 (Armonk, NY: IBM Corp.). Descriptive statistics were performed for all variables. The chi-square test was used to assess the relationship between categorical variables. Statistical significance is considered at the 0.05 alpha level.

Ethical consideration

This study was approved by the International Review Board of King Fahd Hospital, Al-Ahsa, under no. H-05-HS-065, and it was conducted in accordance with the principles outlined in the Declaration of Helsinki. Permission to conduct the study was obtained from managers of PHCs. We informed participants about the study's aim, obtained informed consent from all participants, and maintained confidentiality during data collection. All data were collected anonymously. Participants had the freedom to withdraw without coercion, and no individual information was shared with third parties.

## Results

Participants’ demographics

Table 1 shows that our study included 220 participants, comprised of consultants, specialists, residents, and general practitioners. The mean age of participants was 32.14 years (SD=6.05), with a significant age difference observed across the physician groups (p<0.001); residents were the youngest (mean age 28.14 years), while consultants were the oldest (mean age 38.32 years). More than half of them were males, 118 (53.6%). Regarding years in practice, a significant difference was noted among the groups (p<0.001). More than half of the participants, 130 (59.0%), had practiced for less than five years, largely driven by residents, 73 (96.1%) of whom were in this category. In contrast, the majority of consultants, 26 (63.4%), had more than 10 years of practice. The work setting also varied significantly (p<0.001). While 56.8% of all participants worked in academic centres, 77.1% of general practitioners worked in community settings. Continuing medical education (CME) hours related to diabetes completed in the past three years showed a significant difference (p=0.017). Nearly half of all participants, 97 (44.1%), reported less than five CME hours, while only 68 (30.9%) spent more than 10 hours. Furthermore, 132 (60.0%) of all participants reported no special training in DM in the past three years, a finding that was also significant across groups (p=0.009). Specialists had the highest rate of special training, 32 (58.2%), while general practitioners had the lowest (27.1%).

**Table 1:** Participants’ demographics and clinical practice characteristics (n=220). *Significant p-value. CME: continuing medical education; DM: diabetes mellitus

Categories	Sub-categories	Consultant	Specialist	Resident	General practitioner	All n=220	p-Value
Age	Mean (SD)	38.32 (4.70)	32.0 (4.32)	28.14 (3.29)	33.25 (7.27)	32.14 (6.05)	<0.001
Sex	Male	27 (65.9%)	27 (49.1%)	36 (47.4%)	28 (58.3%)	118 (53.6%)	0.208
	Female	14 (34.1%)	28 (50.9%)	40 (52.6%)	20 (41.7%)	102 (46.4%)	
Years in practice	Less than five years	0 (0.0%)	30 (54.5%)	73 (96.1%)	27 (56.3%)	130 (59.0%)	<0.001
	From five to 10 years	15 (36.6%)	18 (32.7%)	2 (2.6%)	10 (20.8%)	45 (20.5%)	
	More than 10 years	26 (63.4%)	7 (12.7%)	1 (1.3%)	11 (22.9%)	45 (20.5%)	
Setting	Academic center	22 (53.7%)	24 (43.6%)	68 (89.5%)	11 (22.9%)	125 (56.8%)	<0.001
	Community center	19 (46.3%)	31 (56.4%)	8 (10.5%)	37 (77.1%)	95 (43.2%)	
Number of diabetic CME hours completed in the past three years	Less than 5 hours	16 (39.0%)	16 (29.1%)	39 (51.3%)	26 (54.2%)	97 (44.1%)	0.017*
	From 5 to 10 hours	7 (17.1%)	15 (27.3%)	21 (27.6%)	12 (25.0%)	55 (25.0%)	
	More than 10 hours	18 (43.9%)	24 (43.6%)	16 (21.1%)	10 (20.8%)	68 (30.9%)	
Special training in DM in the past three years?	No	27 (65.9%)	23 (41.8%)	47 (61.8%)	35 (72.9%)	132 (60.0%)	0.009*

Attitude and practice toward the prevention of diabetic foot (DF) ulceration in diabetic patients

Table 2 shows the attitudes and practices toward the prevention of diabetic foot (DF) ulceration. More than three-quarters, 153 (69.5%) of physicians, rate the percentage of their diabetic patients whom they have systematically evaluated for risk of diabetic foot (DF) as “less than 50%.” This was particularly pronounced among specialists (p<0.025). For patients with open DF infections, probing for bone exposure was practiced by 102 (46.4%) physicians overall, with no significant difference among the groups (p=0.254). Similarly, 93 (42.3%) of all physicians reported requesting serial plain radiographs for bone abnormalities, with no significant inter-group difference (p=0.659).

**Table 2:** Participants’ understanding, attitude, and practice toward prevention of DF ulceration in diabetic patients (n=220). *Significant p-value. DF: diabetic foot

Categories	Sub-categories, n (%)	Consultant, n (%)	Specialist, n (%)	Resident, n (%)	General practitioner, n (%)	All, n=220	p-Value
What proportion of diabetic patients you have evaluated systematically has a high risk for the diabetic foot?	None	0 (0.0%)	1 (1.8%)	8 (10.5%)	8 (16.7%)	17 (7.7%)	0.025*
	Less than 50%	30 (73.2%)	42 (76.4%)	48 (63.2%)	33 (68.8%)	153 (69.5%)	
	More than 50%	11 (26.8%)	12 (21.8%)	20 (26.3%)	7 (14.6%)	50 (22.7%)	
In patients with diabetic foot infections and open wounds, do you perform probing to check for bone exposure?	No	27 (65.9%)	26 (47.3%)	42 (55.3%)	23 (47.9%)	118 (53.6%)	0.254
	Yes	14 (34.1%)	29 (52.7%)	34 (44.7%)	25 (52.1%)	102 (46.4%)	
	Not sure	0 (0.0%)	0 (0.0%)	0 (0.0%)	0 (0.0%)	0 (0.0%)	
In patients with diabetic foot wounds, do you request a serial plain radiograph of the affected foot to identify any bone abnormality?	No	27 (65.9%)	32 (58.2%)	41 (53.9%)	27 (56.3%)	127 (57.7%)	0.659
	Yes	14 (34.1%)	23 (41.8%)	35 (46.1%)	21 (43.8%)	93 (42.3%)	
	Not sure	0 (0.0%)	0 (0.0%)	0 (0.0%)	0 (0.0%)	0 (0.0%)	
For those patients who require additional imaging, particularly when soft tissue abscess is suspected or the diagnosis of osteomyelitis remains uncertain, what type of imaging will you do?	MRI	38 (92.7%)	48 (87.3%)	59 (77.6%)	28 (58.3%)	173 (78.6%)	<0.001
	CT scan	3 (7.3%)	6 (10.9%)	9 (11.8%)	16 (33.3%)	34 (15.5%)	
	Leukocyte or anti-granulocyte scan	0 (0.0%)	1 (1.8%)	8 (10.5%)	4 (8.3%)	13 (5.9%)	
Do you take wound size measurements of diabetic foot wounds?	No	19 (46.3%)	34 (61.8%)	44 (57.9%)	26 (54.2%)	123 (55.9%)	0.479
	Yes	22 (53.7%)	21 (38.2%)	32 (42.1%)	22 (45.8%)	97 (44.1%)	
How often do you follow up with patients with diabetic foot wounds?	As needed	19 (46.3%)	26 (47.3%)	37 (48.7%)	19 (39.6%)	101 (45.9%)	0.003*
	2 to 3 weeks	13 (31.7%)	25 (45.5%)	26 (34.2%)	15 (31.3%)	79 (35.9%)	
	6 to 8 weeks	7 (17.1%)	1 (1.8%)	8 (10.5%)	2 (4.2%)	18 (8.2%)	
	I don’t follow	2 (4.9%)	3 (5.5%)	5 (6.6%)	12 (25.0%)	22 (10.0%)	
How frequently do you order dressing for patients with diabetic foot wounds?	Daily	19 (46.3%)	29 (52.7%)	31 (40.8%)	24 (50.0%)	103 (46.8%)	0.366
	Every 2 days	15 (36.6%)	10 (18.2%)	18 (23.7%)	10 (20.8%)	53 (24.1%)	
	Twice a day	2 (4.9%)	4 (7.3%)	5 (6.6%)	1 (2.1%)	12 (5.5%)	
	I do not manage wounds	5 (12.2%)	12 (21.8%)	22 (28.9%)	13 (27.1%)	52 (23.6%)	
Where do you order the dressing changes to be done for patients with diabetic foot wounds?	In the primary care center	32 (78.0%)	47 (85.5%)	66 (86.8%)	41 (85.4%)	186 (84.5%)	0.755
	In the hospital	8 (19.5%)	7 (12.7%)	7 (9.2%)	5 (10.4%)	27 (12.3%)	
	At home	1 (2.4%)	1 (1.8%)	3 (3.9%)	2 (4.2%)	7 (3.2%)	
To whom may you refer your patients with diabetic foot wounds? Choose your answer based on your practice.	I sent them to the emergency department	8 (19.5%)	14 (25.5%)	11 (14.5%)	13 (27.1%)	46 (20.9%)	0.558
	General surgeon	25 (61.0%)	27 (49.1%)	42 (55.3%)	21 (43.8%)	115 (52.3%)	
	Vascular surgeon	7 (17.1%)	14 (25.5%)	21 (27.6%)	12 (25.0%)	54 (24.5%)	
	Orthopedic surgeon	1 (2.4%)	0 (0.0%)	2 (2.6%)	2 (4.2%)	5 (2.3%)	

In cases requiring additional imaging for suspected soft tissue abscess or osteomyelitis, MRI was the preferred choice for 78.6% of participants, a preference that differed significantly across groups (p<0.001). For instance, while 38 (92.7%) consultants opted for MRI, only 28 (58.3%) general practitioners did so, with a notable 16 (33.3%) GPs choosing CT scans. Regarding wound size measurements for DF wounds, 97 (44.1%) participants reported taking them, with no significant difference between groups (p=0.479). Follow-up frequency for DF wounds showed a significant difference among professions (p=0.003). While 101 (45.9%) followed up "as needed," 79 (35.9%) followed up every two to three weeks. Notably, 12 (25.0%) of general practitioners stated they "don't follow" these patients. When it came to ordering daily dressings for DF patients, 46.8% did so, while 52 (23.6%) participants stated they "do not manage wounds," with no significant group differences (p=0.366). Most participants, 186 (84.5%), ordered dressing changes to be done in the primary care center, showing no significant variation (p=0.755). Finally, concerning referrals for diabetic foot wounds, the most common referral was to a general surgeon, 115 (52.3%), followed by a vascular surgeon, 54 (24.5%). These referral patterns did not significantly differ among physician groups (p=0.558).

Attitudes and practices toward diagnosis and management of diabetic foot infections

Table 3 highlights distinct patterns in participants' understanding and attitudes toward the diagnosis and management of diabetic foot infections. A significant difference was observed in how participants rated their knowledge of therapies and prevention of diabetic foot disease (p<0.001). While 160 (72.7%) rated their knowledge as average, only 26 (11.8%) considered themselves above average, and 34 (15.5%) rated themselves below average. This below-average self-assessment was particularly high among general practitioners 19 (39.6%). When asked about the recommended interval for foot inspections for diabetic patients, 112 (50.9%) chose every visit. Although consultants had the highest percentage, 25 (61.0%), for this answer, the overall difference across groups was not significant (p=0.077). Most participants, 197 (89.5%), reported educating diabetic patients and their families about preventive foot care, a practice that significantly varied among groups (p=0.012), with residents showing a lower rate, 62 (81.6%), compared to consultants, 40 (97.6%). Regarding advising specialized therapeutic footwear, 106 (48.2%) of participants routinely advised its use, with no significant difference among groups (p=0.307). Furthermore, a majority of 189 (85.9%) physicians recommended wearing specific therapeutic footwear to prevent new or recurrent foot ulcers in high-risk patients with healed DFUs, with consultants leading this practice 38 (92.7%), and no significant difference was found across groups (p=0.327). For recommended adequate glycemic control in diabetic patients, a majority of 203 (93.1%) correctly identified "HbA1c less than 7%," with consultants achieving a 40 (97.6%) correct response rate. This high level of understanding did not show a significant difference among the professional groups (p=0.085). Finally, concerning Ankle-Brachial Index (ABI) measurement for diabetic patients, 88 (40.0%) reported ordering it "when they have non-healing wounds," and 55 (25.0%) ordered it for "all diabetic patients," but no significant difference was found across groups (p=0.376).

**Table 3:** Participants’ understanding, attitudes, and practice toward diagnosis and management of diabetic foot infections in patients with diabetic foot (n=220). *Significant p-value.

Categories	Sub-category	Consultant	Specialist	Resident	General practitioner	All n=220	p- Value
Please rate your knowledge of therapies and the prevention of diabetic foot disease	Below average	2 (4.9%)	3 (5.5%)	10 (13.2%)	19 (39.6%)	34 (15.5%)	<0.001
	Average	30 (73.2%)	43 (78.2%)	63 (82.9%)	24 (50.0%)	160 (72.7%)	
	Above average	9 (22.0%)	9 (16.4%)	3 (3.9%)	5 (10.4%)	26 (11.8%)	
As far as you know, how often should diabetic patients undergo interval foot inspection?	Annually	11 (26.8%)	26 (47.3%)	34 (44.7%)	15 (31.3%)	86 (39.1%)	0.077
	Every 6 months	5 (12.2%)	2 (3.6%)	6 (7.9%)	9 (18.8%)	22 (10.0%)	
	Every visit	25 (61.0%)	27 (49.1%)	36 (47.4%)	24 (50.0%)	112 (50.9%)	
Do you educate the diabetic patients and their families about preventive foot care?	No	1 (2.4%)	2 (3.6%)	14 (18.4%)	6 (12.5%)	23 (10.5%)	0.012*
	Yes	40 (97.6%)	53 (96.4%)	62 (81.6%)	42 (87.5%)	197 (89.5%)	
In your practice, how often do you advise diabetic patients to use specialized therapeutic footwear?	Routinely	21 (51.2%)	29 (52.7%)	36 (47.4%)	20 (41.7%)	106 (48.2%)	0.307
	Only high-risk patients	16 (39.0%)	18 (32.7%)	20 (26.3%)	18 (37.5%)	72 (32.7%)	
	Rarely	4 (9.8%)	8 (14.5%)	20 (26.3%)	10 (20.8%)	42 (19.1%)	
Do you recommend wearing specific therapeutic footwear to aid in the prevention of new or recurrent foot ulcers in high-risk patients with healed diabetic foot ulcers?	No	3 (7.3%)	6 (10.9%)	14 (18.4%)	8 (16.7%)	31 (14.1%)	0.327
	Yes	38 (92.7%)	49 (89.1%)	62 (81.6%)	40 (83.3%)	189 (85.9%)	
	Not sure	0 (0.0%)	0 (0.0%)	0 (0.0%)	0 (0.0%)	0 (0.0%)	
What is the recommended adequate glycemic control in diabetic patients?	HbA1c less than 5%	0 (0.0%)	0 (0.0%)	1 (1.3%)	2 (4.3%)	3 (1.4%)	0.085
	HbA1c less than 6%	1 (2.4%)	0 (0.0%)	7 (9.2%)	4 (8.5%)	12 (5.5%)	
	HbA1c less than 7%	40 (97.6%)	54 (100.0%)	68 (89.5%)	41 (87.2%)	203 (93.1%)	
When do you order an Ankle-Brachial Index (ABI) measurement for diabetic patients?	When they have non-healing wounds	14 (34.1%)	25 (45.5%)	31 (40.8%)	18 (37.5%)	88 (40.0%)	0.376
	You do it to all diabetic patients	12 (29.3%)	14 (25.5%)	16 (21.1%)	13 (27.1%)	55 (25.0%)	
	When they reach 50 years of age	0 (0.0%)	4 (7.3%)	7 (9.2%)	6 (12.5%)	17 (7.7%)	
	When they have wounds	6 (14.6%)	1 (1.8%)	9 (11.8%)	3 (6.3%)	19 (8.6%)	
	Never	9 (22.0%)	11 (20.0%)	13 (17.1%)	8 (16.7%)	41 (18.6%)	

Barriers to the diagnosis and management of diabetic foot infections

Table 4 reveals the perceived barriers to effective diagnosis and management of diabetic foot infections. More than half of the physicians, 137 (64.0%), identified lack of physician knowledge about treating diabetic foot disease as a major barrier (p=0.002), with specialists, 35 (67.3%) and residents, 51 (68.0%), particularly highlighting this. The absence of a vascular medicine specialty in Al-Ahsa was also seen as a barrier by 82 (38.3%) of participants. Lack of diabetic foot management guidelines was another significant concern for 115 (53.7%) of participants, especially specialists 35 (67.3%). Furthermore, lack of continuing education in the importance of diabetic foot disease was cited by 145 (67.8%) of all participants, with specialists 40 (76.9%) and residents 56 (74.7%) emphasizing this barrier.

**Table 4:** Participants’ understanding of barriers toward diagnosis and management of diabetic foot infections in patients with diabetic foot (n=220). *Significant p-value.

Items	Sub-answer	Consultant, n (%)	Specialist, n (%)	Resident, n (%)	General practitioner, n (%)	ALL n=220	p-Value
What is the most important barrier to your diabetic patients not receiving risk-reduction therapies and reaching the target for vascular protection? Multiple answers.							
Lack of knowledge among treating physicians about diabetic foot disease	Yes	25 (62.5%)	35 (67.3%)	51 (68.0%)	26 (55.3%)	137 (64.0%)	0.002*
Absence of a vascular medicine specialty in Al-Ahsa	Yes	22 (55.0%)	23 (44.2%)	28 (37.3%)	9 (19.1%)	82 (38.3%)	
Lack of diabetic foot management guidelines	Yes	23 (57.5%)	35 (67.3%)	36 (48.0%)	21 (44.7%)	115 (53.7%)	
Lack of continuing education on the importance of diabetic foot disease	Yes	23 (57.5%)	40 (76.9%)	56 (74.7%)	26 (55.3%)	145 (67.8%)	
There is a clear pathway to follow in the PHC setting for the management of DM foot	No	24 (58.5%)	32 (58.2%)	30 (39.5%)	16 (33.3%)	102 (46.4%)	<0.001
	Yes	8 (19.5%)	11 (20.0%)	11 (14.5%)	19 (39.6%)	49 (22.3%)	
	Not sure	9 (22.0%)	12 (21.8%)	35 (46.1%)	13 (27.1%)	69 (31.3%)	
I know there is a podiatry clinic to refer Patients with DM foot in Al-Ahsa	No	16 (39.0%)	12 (21.8%)	14 (18.4%)	16 (33.3%)	58 (26.4%)	0.049*
	Yes	22 (53.7%)	31 (56.4%)	40 (52.6%)	20 (41.7%)	113 (51.4%)	
	Not sure	3 (7.3%)	12 (21.8%)	22 (28.9%)	12 (25.0%)	49 (22.2%)	
I know how to refer patients with DM foot to the podiatry clinic	No	11 (26.8%)	17 (30.9%)	36 (47.4%)	20 (41.7%)	84 (38.2%)	0.267
	Yes	20 (48.8%)	24 (43.6%)	24 (31.6%)	15 (31.3%)	83 (37.7%)	
	Not sure	10 (24.4%)	14 (25.5%)	16 (21.1%)	13 (27.1%)	53 (24.1%)	
I have enough tools to examine the feet of DM patients, for example, monofilament, hammer, tuning fork 125	No	22 (53.7%)	37 (67.3%)	51 (67.1%)	30 (62.5%)	140 (63.6%)	0.025*
	Yes	18 (43.9%)	16 (29.1%)	16 (21.1%)	10 (20.8%)	60 (27.3%)	
	Not sure	1 (2.4%)	2 (3.6%)	9 (11.8%)	8 (16.7%)	20 (9.1%)	
I have enough time in my clinic to do a foot examination of diabetic patients	No	28 (68.3%)	44 (80.0%)	52 (68.4%)	28 (58.3%)	152 (69.1%)	0.072
	Yes	12 (29.3%)	5 (9.1%)	16 (21.1%)	12 (25.0%)	45 (20.5%)	
	Not sure	1 (2.4%)	6 (10.9%)	8 (10.5%)	8 (16.7%)	23 (10.5%)	
There is enough equipment for the debridement of a foot ulcer in a DM patient	No	28 (68.3%)	35 (63.6%)	45 (59.2%)	31 (64.6%)	139 (63.2%)	0.462
	Yes	5 (12.2%)	12 (21.8%)	10 (13.2%)	9 (18.8%)	36 (16.4%)	
	Not sure	8 (19.5%)	8 (14.5%)	21 (27.6%)	8 (16.7%)	45 (20.5%)	
There is enough equipment for dressing a foot ulcer in a DM patient	No	19 (46.3%)	23 (41.8%)	21 (27.6%)	18 (37.5%)	81 (36.8%)	0.136
	Yes	14 (34.1%)	24 (43.6%)	29 (38.2%)	20 (41.7%)	87 (39.5%)	
	Not sure	8 (19.5%)	8 (14.5%)	26 (34.2%)	10 (20.8%)	52 (23.6%)	
There is a clear protocol to refer patients with DM foot to a vascular surgeon	No	28 (68.3%)	33 (60.0%)	26 (34.2%)	22 (45.8%)	109 (49.5%)	0.003*
	Yes	6 (14.6%)	7 (12.7%)	13 (17.1%)	12 (25.0%)	38 (17.3%)	
	Not sure	7 (17.1%)	15 (27.3%)	37 (48.7%)	14 (29.2%)	73 (33.2%)	

Regarding the existence of a clear pathway to follow at the primary healthcare center (PHC) setting for DM foot management, there was a significant division (p<0.001). About half of the 102 physicians (46.4%) responded negatively, while only 49 (22.3%) believed that a clear pathway existed. Awareness of a podiatry clinic to refer DM foot patients in Al-Ahsa was significantly different across groups (p=0.049). While 113 (51.4%) physicians were aware of such clinics, 16 (39.0%) consultants were not. Similarly, knowing how to refer patients to a podiatry clinic was not significantly different (p=0.267), with 84 (38.2%) physicians stating that they did not know how. The availability of tools for foot examination (e.g., monofilament, hammer, tuning fork 125) was a significant barrier (p=0.025), with 140 (63.6%) of all participants reporting a lack of such tools, most notably specialists (37 {67.3%}) and residents (51 {67.1%}). More than three-quarters of physicians, 152 (69.1%), reported not having enough time in their clinic to perform comprehensive foot examinations.

Finally, more than half of the physicians, 139 (63.2%), reported a shortage of equipment for debridement of diabetic foot ulcers (DF), and 81 (36.8%) reported a lack of dressing materials; these factors did not show significant inter-group differences. However, the perception of a clear protocol to refer patients with DM foot to a vascular surgeon was significantly different (p=0.003). Almost half, 109 (49.5%), believed there was no clear protocol, with consultants 28 (68.3%) and specialists 33 (60.0%) holding this view more strongly than residents.

## Discussion

Attitude and practice toward the prevention of DF ulceration in diabetic patients

The American Diabetes Association (ADA) recommends performing a comprehensive foot evaluation at least annually to identify risk factors for ulcers and amputations, with patients having high-risk foot conditions requiring more frequent evaluations [12]. The current study stated that 112 (50.9%) participants thought that diabetic patients should undergo interval foot inspection, 86 (39.1%) thought they should undergo it annually, and 10% every six months. A Saudi study by Alsheikh et al. indicated that around two-thirds of their participants (64%) performed inspection for high-risk patients at every visit, 21% annually, and 14% every six months [10]. A study conducted among GPs in European countries showed that diabetic foot ulcer was incidentally diagnosed during routine examination in one-fifth of their cases, indicating the importance of routine examination [13]. The low rate of physical examination may be due to time constraints, lack of training, or underestimation of the importance of these checks.

Additionally, less than half of the participants stated that they perform an inspection for the open wound and expose the bone. Consistently, a study conducted in South Africa revealed that 42% of their nurses examined diabetic patients’ feet for any deformities, calluses, infections, or ulcers at each visit [14]. The International Working Group on the Diabetic Foot recommends annual foot examinations for all patients with diabetes, with more frequent checks for those at higher risk [15]. These evaluations should include assessments for skin integrity, ulceration, infection, and bony involvement [15]. On the other hand, a Saudi study in Riyadh revealed that about 53% of their physicians performed wound probes to determine bone protrusion in an open wound, which is higher than our study [10].

About 103 (46.8%) physicians in the present study stated that they used to order dressing daily for patients with diabetic foot wounds, 12 (5.5%) ordered it twice a day and 53 (24.1%) did it every two days, and 52 (23.6%) stated that they do not manage wounds. Additionally, the majority of physicians, 186 (84.5%), preferred to order the dressing in the primary healthcare centers; 27 (12.3%) preferred to order it in the hospitals, and the rest at home. A study conducted in Qatar found that 58.0% of participants indicated regular use of dressings in the treatment of diabetic foot ulcers, while the preferences for dressings showed a range of choices, with Silvercel being the most commonly utilized at 34.4% [16]. A study conducted in Saudi Arabia revealed that about 18% of their healthcare providers agreed that it is not necessary to address diabetic wounds regularly [17]. This is especially concerning, as undetected ulcers or signs of Charcot foot can lead to serious complications, including infections and amputations [18].

Attitudes and practices toward diagnosis and management of diabetic foot infections

Sensitive neuropathy results in the loss of protective sensation, so the patients don't focus on their initial wound [15]. An effective approach must enhance patient education and awareness to prevent the development of ulcers and facilitate early diagnosis of wounds, thereby reducing the frequency of incidental diagnoses. Overall, the education of patients and the training of practitioners are essential elements in the management of diabetic foot ulcers [19]. In our study, the majority of our participants, 197 (89.5%), indicated that they educate the diabetic patients and their families about preventive foot care. We observed that GPs and resident doctors had lower educational attainment rates compared to consultants and specialist doctors. This education rate was lower than that reported in a Saudi study conducted in Riyadh, which stated that almost all of their doctors (96%) educated their patients about self-inspection; however, the rate among GPs was notably lower [10]. Inconsistently, a study conducted among nurses revealed that 62% used to educate diabetic patients about their feet [14]. This gap between consultants/specialists and GPs/residents is clinically significant. Consultants and specialists work in structured settings focused on chronic disease management, while GPs and residents often face heavy workloads and lack specialized training.

In this study, the majority of physicians recommended wearing specific therapeutic footwear for their diabetic patients to aid in the prevention of new or recurrent foot ulcers. About half of them, 106 (48.2%), advised them routinely; 42 (19.1%) rarely did it, while about one-third, 72 (32.7%), used to advise only high-risk patients. A study conducted among physicians and nurses revealed that all of their participants were knowledgeable about diabetic foot care, providing guidance on self-care and hygiene, appropriate footwear, and the avoidance of detrimental foot care practices [20]. A study conducted in South Africa found that most of its nurses (89%) consult with their patients about diabetes and diabetic foot [14]. Another South African survey study revealed that among medical professionals, 51% concurred that shoes should be fitted and designed to accommodate the shape of the feet, while 22% believed that consultations with doctors are necessary for foot deformities, and 17% expressed a preference for soft and comfortable footwear [21]. Wearing well-fitted shoes can significantly decrease the development of calluses and prevent the formation of toe deformities. This, in turn, helps to minimize the risk of diabetic foot ulcers, which can be a serious complication for patients with diabetes.

Barriers to the diagnosis and management of diabetic foot infections

The most significant barrier preventing diabetic patients from receiving risk-reduction therapies was the lack of continuity in education and knowledge about diabetic foot treatment, as reported by 145 (67.8%) and 137 (64.0%) physicians, respectively; only 88 (40.0%) of these physicians indicated that they had received special training in diabetes management within the past three years. A Chinese study conducted among GPs revealed that 74% of their participants received educational training in the past two years, which is lower than this study [22]. Consistently, a Saudi study stated that 55.2% of their physicians indicated that there is inadequate continuing education regarding diabetic foot, while 51.3% recognized a widespread deficiency in knowledge among the physicians providing treatment [10].

One of the major barriers stated by our population was the absence of clear guidelines and structured pathways for the management of diabetic feet in a PHC setting. Ugwu et al. revealed that the lack of a local diabetes management protocol was recognized by their respondents as the most prevalent challenge encountered in the care of people living with diabetes in their practice [23]. Additionally, some physicians stated there is no clear protocol to refer patients to a vascular surgeon or to podiatric clinics. This problem was noted in a study conducted in the United Kingdom by Normahani et al., where the referral process to specialist services, accessing vascular clinics, and acquiring vascular advice from a multidisciplinary team at a foot clinic were contributing to treatment delays [24].

Time constraints affected more than two-thirds of our physicians, 152 (69.1%), who were unable to examine diabetic foot patients. Many studies conducted in various nations have indicated that insufficient time allocated during consultations with healthcare professionals can lead to the neglect of foot assessments. A study published in Nigeria revealed that patients spent less than 5 minutes in consultations at each foot clinic, which was attributed to a shortage of staff, insufficient resources, and a high patient load [25]. A lack of dedicated time not only compromises the detection of early complications like ulcers and infections but also delays appropriate interventions. For Instance, a systematic review emphasized that delays in the management of diabetic foot conditions are often due to a lack of sufficient consultation time, complicated referral processes, and inadequate initial evaluations by primary healthcare providers, leading to negative consequences such as higher rates of amputation and increased mortality [26].

Lack of resources was one of the major barriers faced by our physicians, in which 152 (69.1%) physicians stated that they do not have enough tools to examine the feet of DM patients, 139 (63.2%) stated they do not have enough equipment for debridement of foot ulcers, 81 (36.8%) stated they do not have enough equipment for dressing of feet, 58 (26.4%) stated there is no podiatric clinic to refer patients to, and 102 (46.4%) stated that there is no clear pathway to follow at the PHC setting for the management of DM foot. These findings are consistent with international literature. A Nigerian study revealed that the problems of care affordability (61%) and poorly equipped health facilities (49%) were the major barriers that physicians faced [23]. A study conducted in the United Kingdom stated that the unavailability of specialized foot care services, which include podiatry, vascular teams, diabetes foot clinics with multiple disciplines, orthotic services, and orthopedic clinics, was the most common barrier identified by health professionals [8]. These systemic deficiencies not only obstruct early diagnosis and efficient management but also elevate the likelihood of avoidable complications, including infection, hospitalization, and amputation.

Strengths and limitations

This study's strengths include its targeted focus on barriers faced by primary care physicians in managing diabetic foot, its use of a valid and pretested questionnaire, its representative random sampling of 220 participants, and its clinical relevance in addressing a significant public health issue. However, the study has limitations, such as its cross-sectional design, which prevents establishing cause-and-effect relationships. It relies on self-reported data, which may introduce recall bias, and its geographical specificity to Al-Ahsa limits generalizability.

## Conclusions

This study highlights significant gaps in knowledge, practice, and resources among healthcare professionals involved in diabetic foot management. While a large majority engage in patient education and recommend appropriate footwear, deficiencies exist in diagnostic practices, such as probing for bone and radiographic imaging, and knowledge regarding therapies and prevention. Furthermore, systemic barriers, including a lack of specialized expertise, clinical guidelines, continuing education, access to tools, and sufficient time for thorough examinations, impede effective care. These findings indicate the importance of targeted interventions to improve clinician competency, such as training workshops, national protocols, and specialist referral systems, which should be implemented. Additionally, it is essential to improve resource availability and tackle systemic barriers to optimize the prevention and management of diabetic foot complications. To advance the field of diabetic foot management, future research should focus on three key areas. First, it is crucial to evaluate targeted interventions like training workshops and national protocols to confirm their effectiveness in improving clinician competency and patient outcomes. Second, a more profound investigation into systemic barriers is needed to understand precisely how factors such as time constraints, limited access to specialized tools, and institutional policies negatively impact the quality of care. Finally, research must explore patient perspectives and adherence to better understand the challenges patients face in following treatment plans, which can inform the development of more effective, patient-centered educational and support programs.

## Appendices

**Table 5:** Participants’ demographics and clinical practice characteristics. CME: continuing medical education; DM: diabetes mellitus

Items	Answers
Age (by years)	
Sex	Male
	Female
Years in practice	Less than 5 years
	From 5 to 10 years
	More than 10 years
Setting	Academic center
	Community center
Number of diabetic CME hours completed in the past three years	Less than 5 hours
	From 5 to 10 hours
	More than 10 hours
Special training in DM in the past 3 years	No
	Yes

**Table 6:** Attitudes and practices toward diagnosis and management of diabetic foot infections.

Items	Answers
What proportion of diabetic patients you have evaluated systematically has a high risk for the diabetic foot?	None
	Less than 50%
	More than 50%
In patients with diabetic foot infections and an open wound, do you perform probing to check for bone exposure?	No
	Yes
	Not sure
In patients with diabetic foot wounds, do you request a serial plain radiograph of the affected foot to identify any bone abnormality?	No
	Yes
	Not sure
For those patients who require additional imaging, particularly when a soft tissue abscess is suspected or the diagnosis of osteomyelitis remains uncertain, what type of imaging will you do?	MRI
	CT scan
	Leukocyte or anti-granulocyte scan
Do you take wound size measurements of diabetic foot wounds?	No
	Yes
How often do you follow-up with patients with diabetic foot wounds?	As needed
	2 to 3 weeks
	6 to 8 weeks
	I don’t follow
How frequently do you order dressing for patients with diabetic foot wounds?	Daily
	Every 2 days
	Twice a day
	I do not manage wounds
Where do you order the dressing changes to be done for patients with diabetic foot wounds?	In the primary care center
	In the hospital
	At home
To whom may you refer your patients with diabetic foot wounds? Choose your answer based on your practice.	I sent them to the emergency department
	General surgeon
	Vascular surgeon
	Orthopedic surgeon

**Table 7:** The attitudes and practices regarding the diagnosis and management of diabetic foot infections.

Items	Answers
Please rate your knowledge of therapies and the prevention of diabetic foot disease	Below average
	Average
	Above average
As far as you know, how often should diabetic patients undergo interval foot inspection?	Annually
	Every 6 months
	Every visit
Do you educate the diabetic patients and their families about preventive foot care?	No
	Yes
In your practice, how often do you advise diabetic patients to use specialized therapeutic footwear?	Routinely
	Only high-risk patients
	Rarely
Do you recommend wearing specific therapeutic footwear to aid in the prevention of new or recurrent foot ulcers in high-risk patients with healed diabetic foot ulcers?	No
	Yes
	Not sure
What is the recommended adequate glycemic control in diabetic patients?	HbA1c less than 5%
	HbA1c less than 6%
	HbA1c less than 7%
When do you order an Ankle-Brachial Index (ABI) measurement for diabetic patients?	When they have non-healing wounds
	You do it to all diabetic patients
	When they reach 50 years of age
	When they have wounds
	Never

**Table 8:** Barriers to the diagnosis and management of diabetic foot infections.

Items	Answers
What is the most important barrier to your diabetic patients not receiving risk-reduction therapies and reaching the target for vascular protection? Multiple answers.	
Lack of knowledge among treating physicians about diabetic foot disease.	Yes/no
Absence of a vascular medicine specialty in Al-Ahsa.	Yes/no
Lack of diabetic foot management guidelines.	Yes/no
Lack of continuing education on the importance of diabetic foot disease.	Yes/no
There is a clear pathway to follow in the PHC setting for the management of DM foot.	No
	Yes
	Not sure
I know there is a podiatric clinic to refer patients with DM foot in Al-Ahsa.	No
	Yes
	Not sure
I know how to refer patients with DM foot to the podiatric clinic.	No
	Yes
	Not sure
I have enough tools to examine the feet of DM patients, for example, monofilament, hammer, and tuning fork 125.	No
	Yes
	Not sure
I have enough time in my clinic to do a foot examination for diabetic patients.	No
	Yes
	Not sure
There is enough equipment for the debridement of a foot ulcer in a DM patient.	No
	Yes
	Not sure
There is enough equipment for dressing a foot ulcer in a DM patient.	No
	Yes
	Not sure
There is a clear protocol to refer patients with DM foot to a vascular surgeon.	No
	Yes
	Not sure

## References

[1] (2014). Diagnosis and classification of diabetes mellitus. Diabetes Care.

[2] Pippitt K, Li M, Gurgle HE (2016). Diabetes mellitus: screening and diagnosis. Am Fam Physician.

[3] International Diabetes Federation (IDF (2025). The diabetes atlas. https://diabetesatlas.org/.

[4] Frykberg RG (2002). Diabetic foot ulcers: pathogenesis and management. Am Fam Physician.

[5] McDermott K, Fang M, Boulton AJ, Selvin E, Hicks CW (2023). Etiology, epidemiology, and disparities in the burden of diabetic foot ulcers. Diabetes Care.

[6] Alshammary S, Othman SA, Alshammari E (2020). Economic impact of diabetic foot ulcers on healthcare in Saudi Arabia: a retrospective study. Ann Saudi Med.

[7] McPherson M, Carroll M, Stewart S (2022). Patient-perceived and practitioner-perceived barriers to accessing foot care services for people with diabetes mellitus: a systematic literature review. J Foot Ankle Res.

[8] Pankhurst CJ, Edmonds ME (2018). Barriers to foot care in patients with diabetes as identified by healthcare professionals. Diabet Med.

[9] Bouillet B, Ahluwalia R, Iacopi E (2021). Characteristics of new patient referrals to specialised diabetic foot units across Europe and factors influencing delays. J Wound Care.

[10] Alsheikh S, AlGhofili H, Alageel R, Ababtain O, Alarify G, Alwehaibi N, Altoijry A (2023). Diabetic foot care: a screening on primary care providers' attitude and practice in Riyadh, Saudi Arabia. Medicina (Kaunas).

[11] Thompson SK (2012). Sampling.

[12] ElSayed NA, Aleppo G, Aroda VR (2023). Improving care and promoting health in populations: standards of care in diabetes-2023. Diabetes Care.

[13] Garcia-Klepzig JL, Sánchez-Ríos JP, Manu C (2018). Perception of diabetic foot ulcers among general practitioners in four European countries: knowledge, skills and urgency. J Wound Care.

[14] Mafusi LG, Egenasi CK, Steinberg WJ, Benedict MO, Habib T, Harmse M, Van Rooyen C (2024). Knowledge, attitudes and practices on diabetic foot care among nurses in Kimberley, South Africa. S Afr Fam Pract (2004).

[15] Bakker K, Apelqvist J, Schaper NC (2012). Practical guidelines on the management and prevention of the diabetic foot 2011. Diabetes Metab Res Rev.

[16] Waheed MA, Singh K, Al-Lenjawi B (2024). Primary care physicians’ knowledge, attitude, and practice towards diabetic foot ulcer at primary healthcare corporation, Qatar: a cross-sectional survey. Sch J App Med Sci.

[17] Alsaigh SH, Alzaghran RH, Alahmari DA, Hameed LN, Alfurayh KM, Alaql KB (2022). Knowledge, awareness, and practice related to diabetic foot ulcer among healthcare workers and diabetic patients and their relatives in Saudi Arabia: a cross-sectional study. Cureus.

[18] Rogers LC, Frykberg RG, Armstrong DG (2011). The Charcot foot in diabetes. Diabetes Care.

[19] Schaper NC, van Netten JJ, Apelqvist J, Bus SA, Hinchliffe RJ, Lipsky BA (2020). Practical guidelines on the prevention and management of diabetic foot disease (IWGDF 2019 update). Diabetes Metab Res Rev.

[20] Saverio S, Mohammadnezhad M, Raikanikoda F (2024). Healthcare workers' perspectives on diabetic foot complications among type 2 diabetes mellitus patients in Fiji. PLoS One.

[21] Mukheli T, Fourie A, Mokoena TP, Kagodora SB, Luvhengo TE (2025). Medical doctors, nurses, and therapeutic health practitioners knowledge of risk factors and prevention of diabetic foot ulcer: a cross-sectional survey in a South African setting. Diabetology.

[22] Zhao N, Xu J, Zhou Q (2023). Screening behaviors for diabetic foot risk and their influencing factors among general practitioners: a cross-sectional study in Changsha, China. BMC Prim Care.

[23] Ugwu E, Young E, Nkpozi M (2020). Diabetes care knowledge and practice among primary care physicians in Southeast Nigeria: a cross-sectional study. BMC Fam Pract.

[24] Normahani P, Mustafa C, Standfield NJ, Duguid C, Fox M, Jaffer U (2018). Management of peripheral arterial disease in diabetes: a national survey of podiatry practice in the United Kingdom. J Foot Ankle Res.

[25] Ekore RI, Ajayi IO, Arije A, Ekore JO (2010). Knowledge of and attitude to foot care amongst type 2 diabetes patients attending a university-based primary care clinic in Nigeria. Afr J Prim Health Care Fam Med.

[26] Lingyan L, Liwei X, Han Z (2024). Identification, influencing factors and outcomes of time delays in the management pathway of diabetic foot: a systematic review. J Tissue Viability.

